# Aggrecanolysis and *in vitro *matrix degradation in the immature bovine meniscus: mechanisms and functional implications

**DOI:** 10.1186/ar2862

**Published:** 2009-11-17

**Authors:** Christopher G Wilson, Eric J Vanderploeg, Fengrong Zuo, John D Sandy, Marc E Levenston

**Affiliations:** 1 Wallace H Coulter Department of Biomedical Engineering, 313 Ferst Drive, Georgia Institute of Technology, Atlanta, GA 30332, USA; 2 George W Woodruff School of Mechanical Engineering, Georgia Institute of Technology, 801 Ferst Drive, Atlanta, GA 30332, USA; 3 Roche-Palo Alto, 3431 Hillview Avenue, Palo Alto, CA 94304, USA; 4 Department of Biochemistry, Rush University Medical Center, 1735 West Harrison Street, 5th Floor Cohn Building, Chicago, IL 60612, USA

## Abstract

**Introduction:**

Little is known about endogenous or cytokine-stimulated aggrecan catabolism in the meniscal fibrocartilage of the knee. The objectives of this study were to characterize the structure, distribution, and processing of aggrecan in menisci from immature bovines, and to identify mechanisms of extracellular matrix degradation that lead to changes in the mechanical properties of meniscal fibrocartilage.

**Methods:**

Aggrecanase activity in the native immature bovine meniscus was examined by immunolocalization of the aggrecan NITEGE neoepitope. To investigate mechanisms of cytokine-induced aggrecan catabolism in this tissue, explants were treated with interleukin-1α (IL-1) in the absence or presence of selective or broad spectrum metalloproteinase inhibitors. The sulfated glycosaminoglycan (sGAG) and collagen contents of explants and culture media were quantified by biochemical methods, and aggrecan catabolism was examined by Western analysis of aggrecan fragments. The mechanical properties of explants were determined by dynamic compression and shear tests.

**Results:**

The aggrecanase-generated NITEGE neoepitope was preferentially localized in the middle and outer regions of freshly isolated immature bovine menisci, where sGAG density was lowest and blood vessels were present. In vitro treatment of explants with IL-1 triggered the accumulation of NITEGE in the inner and middle regions. Middle region explants stimulated with IL-1 exhibited substantial decreases in sGAG content, collagen content, and mechanical properties. A broad spectrum metalloproteinase inhibitor significantly reduced sGAG loss, abrogated collagen degradation, and preserved tissue mechanical properties. In contrast, an inhibitor selective for ADAMTS-4 and ADAMTS-5 was least effective at blocking IL-1-induced matrix catabolism and loss of mechanical properties.

**Conclusions:**

Aggrecanase-mediated aggrecanolysis, typical of degenerative articular cartilage, may play a physiologic role in the development of the immature bovine meniscus. IL-1-induced release of sGAG and loss of mechanical properties can be ascribed primarily to the activity of MMPs or aggrecanases other than ADAMTS-4 and ADAMTS-5. These results may have implications for the clinical management of osteoarthritis.

## Introduction

The knee menisci have essential roles to play in load transfer and distribution during joint motion, and partial or complete meniscectomy initiates degeneration of the adjacent articular cartilage [1,2]. Meniscal fibrocartilage is rich in circumferentially- and radially-oriented collagen fibrils [3] and extracellular matrix (ECM) proteoglycans including aggrecan, decorin, and biglycan [4]. In articular cartilage, aggrecan confers compressive and shear stiffness through its attached sulfated glycosaminoglycan (sGAG) chains [5,6]. Valiyaveettil and colleagues immunolocalized the G1 domain of aggrecan (the globular hyaluronan-binding domain) between collagen fibrils in the canine meniscus and proposed that aggrecan dissipates compressive loads in the meniscus [7]. Other fibrocartilages, such as regions of deep flexor tendon, are also enriched in high molecular weight aggrecan [8].

Meniscal cells exhibit regional differences in morphology and ECM metabolism [9]. While having similar rates of collagen synthesis, cells from the inner region of the meniscus exhibit higher sGAG accumulation rates than cells from the outer region [10,11]. Regional variations in meniscus sGAG content apparently result from differences in aggrecan concentration due in part to regional differences in aggrecan gene expression [7,12]. Whereas the molecular weight of the full-length aggrecan core protein is about 450 kDa, an abundance of 66 to 70 kDa-sized aggrecan fragments was identified in extracts of bovine meniscal fibrocartilage, suggesting that extensive aggrecan cleavage is normal in this tissue [13]. The size and C-terminal neoepitope (NITEGE, a peptide sequence exposed upon proteolytic cleavage) of those fragments indicated that aggrecanases of the a disintegrin and metalloproteinase with thrombospondin motifs (ADAMTS) family were responsible for the observed aggrecan processing [14,15]. Significantly, the NITEGE neoepitope was also immunolocalized in the menisci of fetal human joints [16], suggesting that aggrecanolysis during development of the menisci is conserved across species. The implications of this extensive aggrecan cleavage in the normal immature meniscus are unknown, and regional differences in the aggrecanase activity of meniscal fibrocartilage have not yet been described.

The proinflammatory cytokines IL-1α and β are linked to the onset of arthritis and initiate aggrecanolysis followed by collagen degradation in articular cartilage [17,18]. *In vivo*, complete loss of sGAG and the onset of collagen damage appear to mark a degenerative 'point of no return' [19-21]. In IL-1-stimulated bovine articular cartilage explants, aggrecanolysis and the associated depletion of sGAG are mediated by ADAMTS-4 and/or -5 (aggrecanase-1 and -2, respectively) and lead to loss of tissue mechanical properties [22]. ADAMTS-5 knock-out mice exhibit profound resistance to ECM resorption in *in vivo *models of osteoarthritis [23,24], and pharmacologic inhibitors of aggrecanases and MMPs can delay or reduce matrix destruction in IL-1-stimulated bovine articular cartilage [25-27]. There are few detailed reports describing the response of fibrocartilage to IL-1 stimulation or the participation of aggrecanases in the remodeling of fibrocartilage ECM. IL-1 treatment of explanted rabbit menisci increased nitric oxide and MMP production [28], and cells from fibrocartilage of the rat temporomandibular joint exhibited upregulated expression of MMPs in the presence of IL-1β [29]. IL-1 abrogated the biosynthetic response of porcine meniscal cells to mechanical stimuli [30]. Both IL-1 and TNF-alpha were recently shown to inhibit the intrinsic repair capacity of explanted meniscal fibrocartilage, and pharmacologic inhibition of MMPs partially rescued the repair response [31-34]. However, neither the enzymatic mechanisms responsible for sGAG release in IL-1-stimulated fibrocartilage nor the IL-1-induced changes in fibrocartilage mechanical properties have been previously reported.

The objective of the current study was to identify mechanisms of aggrecan catabolism in freshly isolated and IL-1-stimulated immature bovine meniscus. The enzymatic activities of aggrecanases and MMPs were perturbed using pharmacologic inhibitors, and tissue degradation was assessed using biochemical assays, western blots, and mechanical tests. The results indicate that the NITEGE neoepitope accumulated preferentially in the middle and outer regions of the immature meniscus, where the sGAG density was lowest and blood vessels were readily detected. IL-1 stimulation of meniscus explants caused a predominantly matrix metalloproteinase (MMP)-mediated release of sGAG and loss of mechanical properties. Collectively, the data indicate that regional variations in generation of the NITEGE neoepitope is normal in the developing immature bovine meniscus and cytokine-induced degradation of the meniscus is mediated primarily by MMPs.

## Materials and methods

### Reagents and antibodies

DMEM, gentamicin, non-essential amino acids (NEAA), N-(2-hydroxyethyl)-piperazine-N'-2-ethanesulfonic acid (HEPES), trypsin-ethylenediaminetetraacetic acid, proteinase K, PBS and Alexafluor 488 anti-rabbit and Alexafluor 594 anti-mouse secondary antibodies were from Invitrogen (Carlsbad, CA, USA). Antibodies to aggrecan G1 and the NITEGE neoepitope were prepared as previously described [35]. The antibody to type I collagen was from Abcam (Cambridge, MA, USA), and the antibody to bovine decorin (LF-94) was a gift from Dr. Larry Fischer (National Institute of Dental and Craniofacial Research, Bethesda, MD, USA). Fluorescein isothiocyanate (FITC)-conjugated secondary antibody was from Chemicon (Temecula, CA, USA). Guanidine hydrochloride, chondroitinase ABC, keratanase I, chloramine-T, para-(dimethylamino)-benzaldehyde (pDAB), non-immune rabbit IgG, alkaline phosphatase-conjugated secondary antibody, 4',6-diamidino-2-phenylindole dihydrochloride (DAPI), and other histologic reagents were from Sigma (St. Louis, MO, USA). Verhoeff's stain was from Poly Scientific (Bay Shore, NY, USA). Keratinase II was from Associates of Cape Cod (Falmouth, MA, USA). Dimethylmethylene blue (DMMB) was from Polysciences (Warrington, PA, USA). Recombinant human IL-1α (*rh*IL-1α) was from Peprotech (Rocky Hill, NJ, USA).

Protease inhibitor cocktail I was from Calbiochem (San Diego, CA, USA). The enhanced chemifluorescence (ECF) substrate was from Amersham (Piscataway, NJ, USA). Metalloproteinase inhibitors (RO3310769, aggrecanase-1,2-selective; RO1136222, MMP-selective; RO4002855, broad-spectrum metalloproteinase) with previously described selectivity profiles [27] were provided by Roche - Palo Alto (Palo Alto, CA, USA).

### Histology and immunostaining

**Figure 1:**
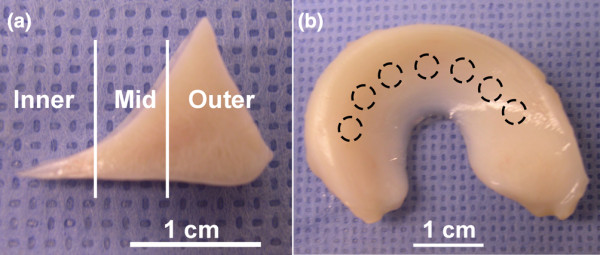
Immature bovine menisci. **(a) **Coronal sections were harvested from immature bovine menisci for histologic and immunofluorescence analysis and the preliminary tissue culture experiments. **(b) **Cylindrical explants from the middle region were prepared for the inhibitor study.

Immature (one to two weeks) bovine menisci (Research 87, Cambridge, MA, USA) were harvested aseptically within 24 hours of slaughter. Coronal 'slabs' (3 to 4 mm thick, Figure 1a) were either fixed in 10% neutral-buffered formalin (NBF) at 4°C for 48 hours and embedded in paraffin or fixed in 10% NBF for four hours at 4°C, incubated in 30% sucrose/PBS overnight at 4°C, embedded in optimal cutting temperature (OCT) embedding medium, and frozen in liquid-nitrogen cooled isopentane. Paraffin-embedded tissue was sectioned at 3 μm, stained for sGAG with safranin-O, and counterstained with fast green and hemotoxylin. Blood vessels were identified in sections stained with Verhoeff's elastin stain, differentiated in 2% ferric chloride, rinsed in 5% sodium thiosulfate, and counterstained with van Gieson's collagen stain. Frozen tissue was sectioned at 7 μm and co-immunostained for the aggrecan NITEGE neoepitope and collagen type I. Sections were digested with 0.5% trypsin for 10 minutes at 37°C and blocked for one hour at room temperature with blocking buffer containing 2% goat serum, 0.1% gelatin, 1% bovine serum albumin, 0.1% Triton, and 0.05% Tween-20 in PBS prior to incubation with antibodies. Primary antibodies were detected by indirect immunofluoresence with Alexafluor 488- and Alexafluor 594-conjugated secondary antibodies. Following tissue culture, 3 to 4 mm thick coronal slabs were fixed in 10% NBF for 48 hours at 4°C, embedded in paraffin, and sectioned at 3 μm. The NITEGE neoepitope was detected by indirect immunofluoresence with a goat anti-rabbit FITC-conjugated secondary antibody. All antibodies were used at 10 μg/mL with incubations for 60 to 90 minutes at room temperature. Cell nuclei were stained with DAPI.

Species-matched non-immune IgGs were used as negative controls. Images of histochemically stained sections were captured on a Nikon E600 microscope (Nikon USA, Melville, NY, USA). Immunofluorescent sections were imaged on a Zeiss AxioVert 200 M microscope using the Apotome optical sectioning module (Zeiss, Jena, Germany).

### Tissue culture

In a preliminary study, coronal sections (Figure 1a) were dissected from immature bovine menisci and cultured in six-well plates with or without 20 ng/mL *rh*IL-1α in serum-free media consisting of DMEM, 10 mM NEAA, 50 μg/mL gentamicin, and 50 μg/mL ascorbic acid under standard conditions (5% CO_2_, 95% humidity and 37°C). Media were exchanged after 48 hours. After four days, the tissue was fixed and processed for immunofluorescent detection of the NITEGE neoepitope.

Full-thickness cylindrical specimens (3 mm in diameter) were harvested from the middle region of menisci and cut to 2 mm in thickness with superficial tissue excluded (Figure 1b), generating samples with well-controlled geometries and low variability in initial wet mass (16.1 ± 1.7 mg). Explants were pre-cultured for 72 hours in 0.5 mL of 'basal' serum-free media consisting of DMEM, 10 mM NEAA, 50 μg/mL gentamicin, and 50 μg/mL ascorbic acid, followed by up to 12 days with or without 20 ng/mL *rh*IL-1α. Some explants were additionally treated with 20 μM of the aggrecanase-selective inhibitor, 5 μM of the MMP-selective inhibitor, or 5 μM of the broad-spectrum metalloproteinase inhibitor (doses based on previous studies [27]). Media were exchanged every 48 hours. Explants (n = 6 per group) were collected at days 0, 4, 8, and 12 days of culture and stored frozen in PBS with protease inhibitors. Day 0 and 8 explants were lyophilized and extracted for 48 hours at 4°C in 10 volumes of extraction buffer (4 M guanidine hydrochloride, 10 mM 2-(N-morpholino)ethanesulfonic acid, 50 mM sodium acetate, 5 mM ethylenediaminetetraacetic acid, 5 mM iodoacetic acid at pH 6.5) with protease inhibitors.

### Mechanical testing

Day 0 and day 12 explants were thawed, weighed, and measured using digital calipers. All tests were performed in PBS with protease inhibitors after compression by 10% of the explant thickness and 12 minutes of stress relaxation. Explants were tested in torsional shear on a CVO120 rheometer (Bohlin, East Brunskwick, NJ, USA) with a 0.5% nominal shear strain at 0.001 to 0.1 Hz to determine dynamic shear moduli G*. Following equilibration to the free-swelling state, the samples were recompressed and tested in oscillatory unconfined compression on an ELF3200 (Enduratec, Minnetonka, MN, USA) with a 1.5% strain amplitude at 0.001 to 0.1 Hz to determine the dynamic compressive moduli E*.

### Biochemistry

Explants from day 0, 4, and 12 were lyophilized, weighed, and digested in 0.0125 mg/mg tissue proteinase K. Explant digests, explant extracts, and conditioned media were assayed for sGAG content by the DMMB dye-binding method [36]. Digests and conditioned media were assayed for hydroxyproline content by the chloramine-T/pDAB reaction [37], with collagen content calculated by assuming a collagen:hydroxyproline mass ratio of 8:1 [38].

### Western blotting

The inner (about 5 mm), middle (about 5 mm), and outer (about 8 mm; along lines drawn in Figure 1a) of immature bovine menisci were dissected, minced, and extracted in extraction buffer (80 mg wet tissue per ml) for 48 hours at 4°C. Conditioned media from the inhibitor study were pooled from days 2 and 4, 6 and 8, or 10 and 12 (from six explants per condition), and equal portions of day 0 and day 8 explant extracts were pooled (six explants per condition and time point). Proteoglycans were isolated from the media and extracts by ethanol precipitation, dried, and deglycosylated by overnight digestion in chondroitinase ABC and keratanases I and II. Equal volumes of conditioned media or native tissue extracts, or equal quantities of sGAG (5 μg for explant extracts), were separated by electrophoresis and transferred to nitrocellulose. Blotting equal volumes of media or tissue extract afforded direct comparison of the abundance per wet weight of the proteins of interest. Blotting equal amounts of sGAG (as with explant extracts) allowed comparison of the abundance of sGAG-bearing proteins per μg total sGAG. Following blocking, membranes were probed with primary antibodies at 1 μg/mL. Blots were developed using an alkaline phosphatase secondary antibody and exposure to ECF. Bands were visualized using a Fuji FLA3000 (Fuji, Tokyo, Japan).

### Statistics

The main effects of inhibitor treatments (at each time point) were evaluated using a general linear model in MINITAB Release 14 (MINITAB, States College, PA, USA). Pairwise comparisons between groups were determined by Dunnett's test with IL-1-only treated samples as controls and significance at *P *< 0.05.

## Results

### Regional variations in sGAG content and the aggrecanase-generated NITEGE neoepitope in the immature meniscus

**Figure 2:**
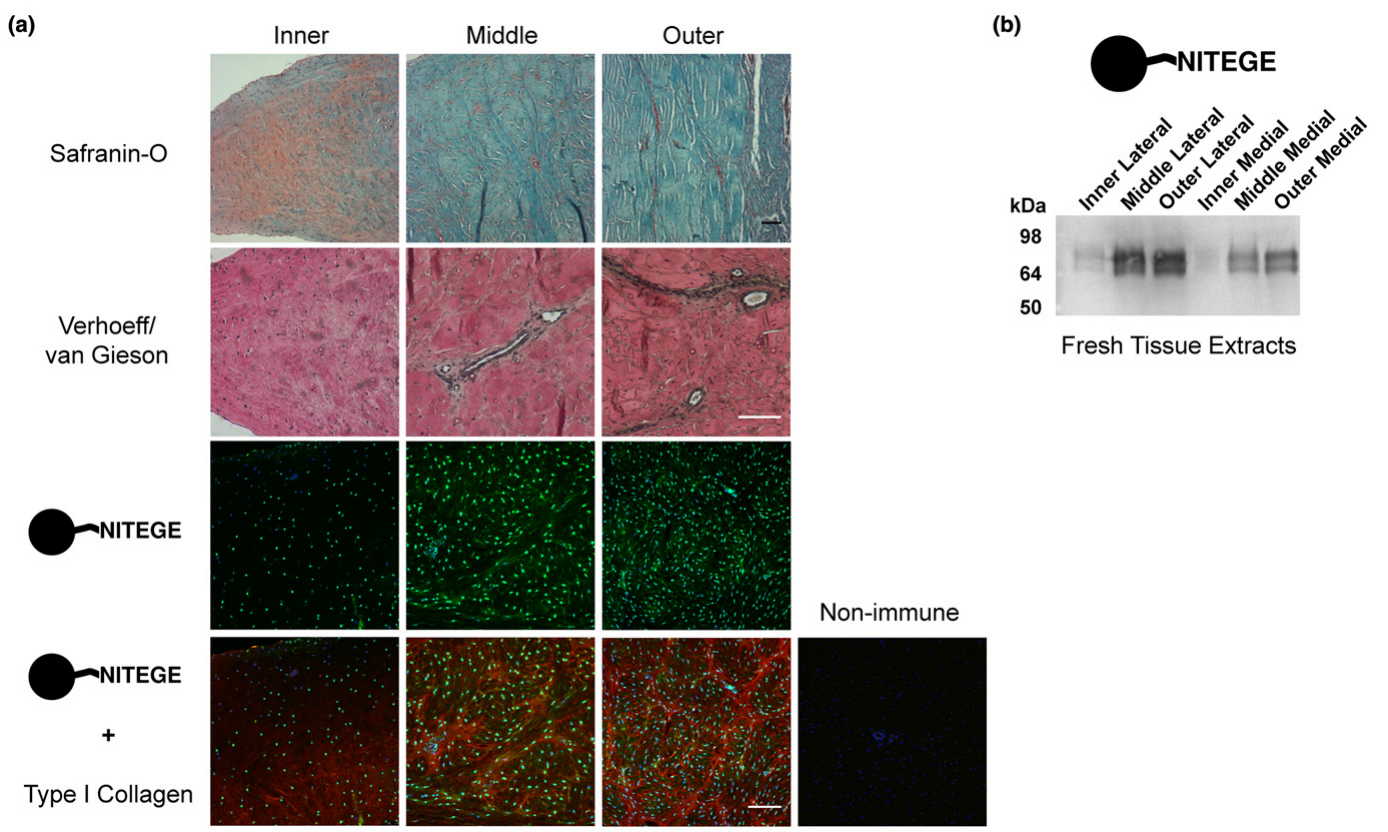
Regional variations in proteoglycan content, vasculature, and aggrecanase product abundance (G1-NITEGE) in freshly isolated immature bovine meniscus. **(a) **Coronal sections of medial meniscus stained for sulfated glycosaminoglycan (red = safranin-O stain; counterstained with fast green), elastin (black = Verhoeff's stain; pink = van Gieson's stain for collagen), or the aggrecan NITEGE neoepitope (green) and collagen type I (red). In immunofluorescent images, the cell nuclei are blue. Inner, middle, and outer refer to the radial position in the coronal plane. Scale bars = 100 μm. **(b) **The regional variations in aggrecan NITEGE abundance were confirmed by western blot of tissue extracts of both lateral (left lanes) and medial (right lanes) menisci. Equal volumes of tissue extracts (each from about 5 mg wet mass tissue) were loaded in each lane. The migration of globular protein standards are shown at left.

Safranin-O staining revealed marked regional variations in sGAG content within the immature meniscus with the most intense staining within the inner region (Figure 2a). There were also sGAG-rich pockets in the middle and outer regions colocalized with cells and between collagen fibril bundles. These patterns are consistent with previously reported regional variations in canine meniscus aggrecan content [7], porcine aggrecan gene expression [12], and ovine proteoglycan synthesis rates [39]. Verhoeff's stain demonstrated the presence of blood vessels in the middle and outer regions of the immature meniscus. Vessels were identified by elastin staining (black) and the presence of open lumens. The inner region was consistently devoid of blood vessels.

The abundance of the aggrecan NITEGE neoepitope, a product of aggrecanase activity, varied across coronal sections of the immature meniscus (Figure 2a, lower panels), with weak staining in the inner region but intense intra- and peri-cellular staining in the middle and outer regions. The middle and outer regions also exhibited NITEGE staining along and within collagen fibril bundles. NITEGE appeared in regions where safranin-O staining was weak and blood vessels were present. Immunoblots of tissue extracts (Figure 2b) supported the staining patterns, demonstrating regional variations in the abundance of NITEGE in both medial and lateral menisci.

### Localization of the NITEGE neoepitope in IL-1-stimulated meniscal fibrocartilage

**Figure 3:**
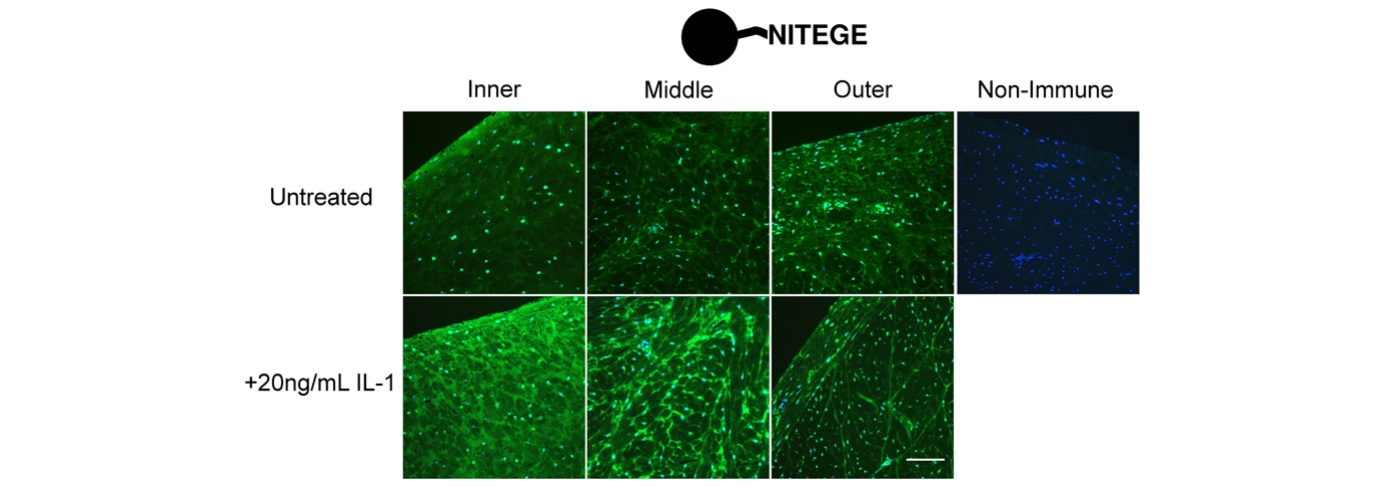
Localization of the NITEGE neoepitope in immature meniscus in the absence and presence of IL-1. Aggrecan NITEGE (green) detected in untreated (top) and IL-1-stimulated (bottom) meniscal fibrocartilage. Cell nuclei are blue. Inner, middle, and outer refer to the radial position in the coronal plane. Scale bar = 100 μm.

When cultured coronal sections of meniscus were examined by immunohistochemistry, the unstimulated controls showed regional variations in NITEGE abundance similar to freshly isolated tissue and an increase in staining in the surface zone of the inner region (Figure 3). In contrast, IL-1-stimulated tissue exhibited marked increases in NITEGE staining throughout the inner and middle regions. The middle region of IL-1-stimulated meniscus demonstrated enhanced NITEGE staining around fibril bundles, seen as a brightly stained fine network that was less intense in the inner region and absent from the outer region. These data indicate that IL-1 stimulation of immature meniscal fibrocartilage triggers the exposure of the NITEGE neoepitope, presumably through aggrecanase-mediated degradation of aggrecan.

### IL-1-induced release of sGAG and aggrecan processing in the immature meniscus

**Figure 4:**
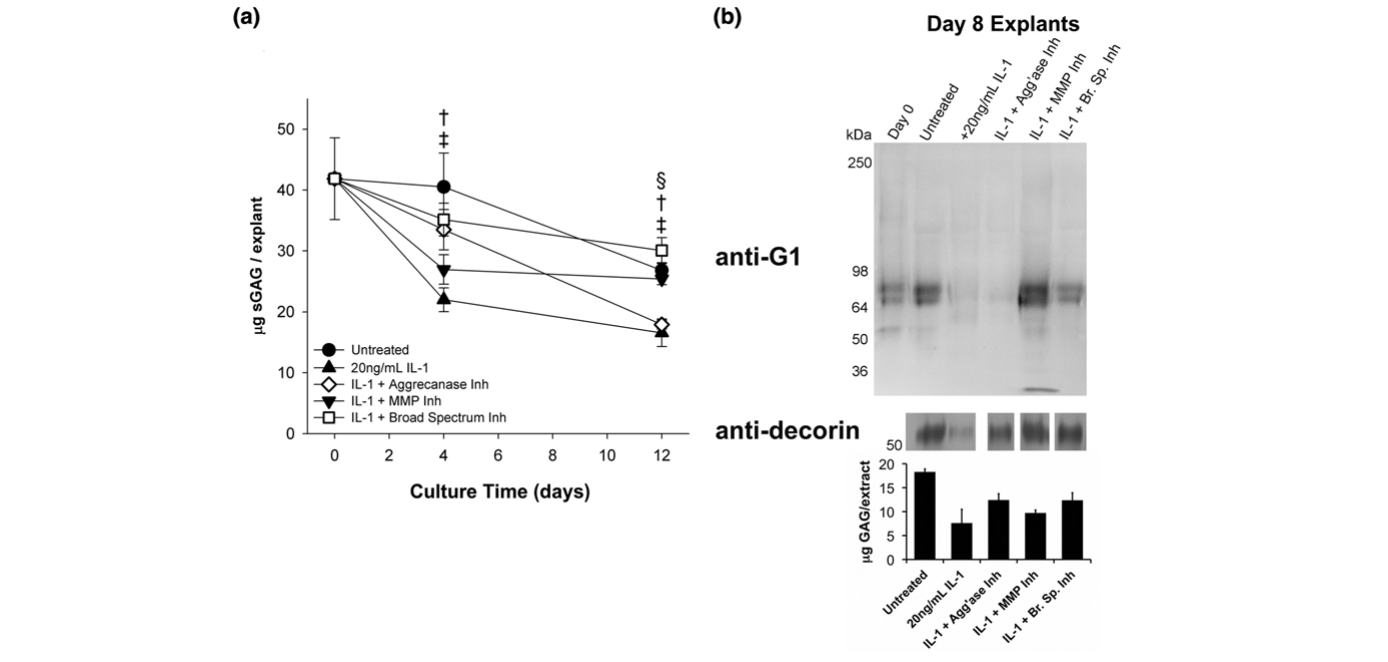
Inhibition of IL-1-induced proteoglycan depletion in immature meniscal fibrocartilage. **(a) **Sulfated glycosaminoglycan (sGAG) contents of middle-zone meniscal fibrocartilage explants. § †, and ‡ indicate *P *< 0.05 vs. IL-1 for IL-1 + matrix metalloproteinases (MMP) inhibitor, IL-1 + broad-spectrum inhibitor, and untreated, respectively. **(b) **Aggrecan G1 fragments and decorin core protein in day 0 and day 8 explants. A 5 μg sample of extracted sGAG, pooled from six explants per condition, were loaded in each lane. The doublet migrating at about 65 kDa was confirmed as the G1-NITEGE product (not shown). The migration of globular protein standards are shown at left. Below blots are sGAG contents of the explant extracts from day 8.

IL-1 stimulation triggered significant, time-dependent reductions in the sGAG content of meniscal explants (Figure 4a). Inhibition of aggrecanase-1 and -2 did not significantly enhance sGAG retention relative to IL-1-only controls at either day 4 or 12. Conversely, explants treated with an MMP-selective inhibitor exhibited sGAG depletion similar to IL-1-only controls over days 0 to 4 but had significantly higher sGAG contents than IL-1-only controls at day 12. Explants treated with a broad-spectrum inhibitor exhibited a blunted acute IL-1-induced release of sGAG and maintained significantly higher sGAG contents than IL-1-only controls at days 4 and 12. These data suggest that aggrecanases-1 and -2 participate in the early release of sGAG in response to IL-1, but MMPs and/or other aggrecanases contribute substantially to the release of sGAG later in the degradative process.

The size and structure of aggrecan in meniscal explants from day 0 and 8 were examined by western blot for the aggrecan G1 domain (Figure 4b). The sGAG contents of day 8 explant extracts (Figure 4b, lower panel) are provided for reference and are consistent with the temporal trajectories of explant digest sGAG content depicted in Figure 4a. A doublet migrating at 66 to 70 kDa was an abundant product in explant extracts and confirmed as the G1-NITEGE fragment in blots for the NITEGE neoepitope (not shown). The blot reveals a paucity of high molecular weight aggrecan at day 0 (and later culture times), although this may be attributable to relatively poor extraction of sGAG-bearing proteoglycans from the explants. We previously found that (with the guanidine extraction buffer used in the present study) proteoglycans are more difficult to extract from meniscal fibrocartilage than from age- and species-matched articular cartilage [40]. Over the course of eight days in culture, the abundance of 66 to 70 kDa aggrecan increased slightly in untreated explants and was markedly reduced in IL-1-treated tissue. The aggrecanase-1,2-selective inhibitor had little effect on the IL-1-induced depletion of this species, whereas both the MMP-selective and broad-spectrum inhibitors abrogated this effect of IL-1 on the explants, suggesting that MMPs mediate the release of aggrecan-G1 from IL-1-stimulated meniscal fibrocartilage.

We also examined the abundance of the small leucine-rich proteoglycan decorin in the explant extracts (Figure 4b, middle panel). Decorin was readily detected in untreated controls but was almost absent in IL-1-stimulated explants. Interestingly, treatment with any of the metalloproteinase inhibitors abolished the IL-1-induced depletion of decorin. These results underscore the susceptibility of other proteoglycans to IL-1-induced ECM catabolism, and suggest that some sGAG released from the explants is associated with non-aggrecan proteoglycans.

**Figure 5:**
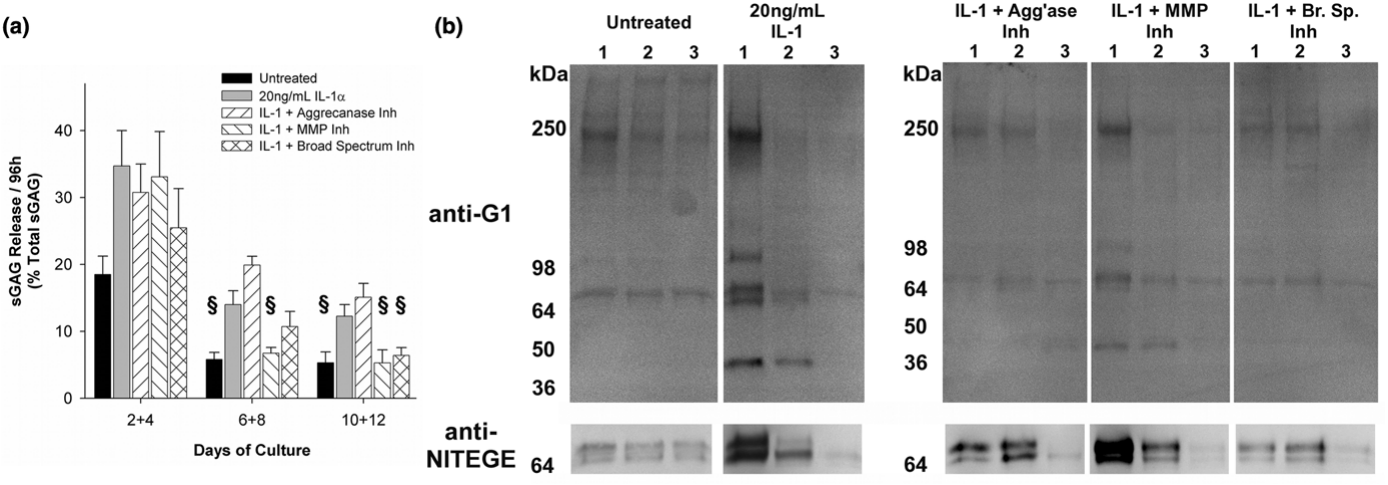
Sulfated glycosaminoglycan (sGAG) and aggrecan release from meniscal fibrocartilage to conditioned media. **(a) **Media from day 12 explants were pooled by time points (days 2 + 4, 6 + 8, and 10 + 12) and assayed for sGAG by the dimethylmethylene blue (DMMB) assay. Data are mean + standard error of the mean with n = 6. § indicates *P *< 0.05 vs. IL-1. **(b) **Media were then blotted for aggrecan G1 fragments and NITEGE neoepitope. Equal volumes of media, pooled from six explants per condition, were loaded in each lane. Lane 1 = media pooled from days 2 + 4; Lane 2 = media pooled from days 6 + 8; Lane 3 = media pooled from days 10 + 12. Migration of protein standards are shown at left. In lower panels, arrowheads indicate migration of the 64 kDa molecular weight marker. Note that the early sGAG release data from day 12 explants should not be quantitatively compared with the day 4 residual sGAG contents (Figure 4a) from different explants. MMP = matrix metalloproteinases.

Assays of conditioned media confirmed that IL-1 stimulated the release of sGAG and aggrecan from meniscal fibrocartilage (Figure 5). Release of sGAG peaked early in the experiment (days 0 to 4) and was substantially lower at later timepoints (Figure 5a). The amount of sGAG released from unstimulated controls was about 50% of that released from IL-1-treated explants. None of the inhibitor treatments significantly reduced IL-1-induced sGAG release over the first 96 hours, but treatment with the MMP-selective or broad-spectrum inhibitors significantly reduced sGAG in the media at later time points.

Using antibodies to the G1 domain and the NITEGE neoepitope, various aggrecan fragments were detected in the conditioned media (Figure 5b). Untreated controls released high molecular weight species at about 400 kDa (full-length aggrecan), about 280 kDa, and about 250 kDa as well as a low molecular weight species migrating at about 70 kDa and bearing the NITEGE neoepitope (Figure 5b, lower panels). Media from IL-1-stimulated samples contained the 250 kDa G1 species and the 66 to 70 kDa NITEGE doublet found in media from unstimulated controls, as well as additional G1 species migrating at about 100 kDa and about 45 kDa. Previous work with the anti-G1 antibody has shown that the 45 kDa band is due to reactivity with link protein [15]. The bulk of aggrecan G1 was released from IL-1-stimulated fibrocartilage within the first 96 hours, when a large fraction of sGAG was released to the media. Treatment with the aggrecanase-1,2-selective inhibitor diminished the IL-1-induced release of the 250 kDa G1 species and NITEGE-bearing fragments to media over the first 96 hours, and abolished release of the 100 kDa and 45 kDa G1 species. The MMP-selective inhibitor also appeared to diminish aggrecan release to the media, but this inhibitor did not block release of the 100 kDa or 45 kDa species and release of NITEGE was similar to IL-1-only controls. Significantly, the broad-spectrum inhibitor restored the temporal pattern of aggrecan (G1 and NITEGE) release in IL-1-stimulated explants to that of unstimulated controls.

### Inhibition of IL-1-induced collagen degradation in the immature meniscus

**Figure 6:**
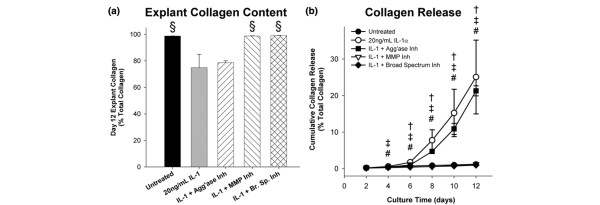
Inhibition of IL-1-induced collagen (a) release to media and (b) loss from immature middle-zone meniscal explants by metalloproteinase inhibitors. Data are mean ± standard error of the mean with n = 6. †, ‡, and # indicate *P *< 0.05 vs. IL-1 for untreated, IL-1 + matrix metalloproteinase (MMP) inhibitors, and IL-1 + broad-spectrum inhibitors, respectively.

Assays of explant digests demonstrated that IL-1 triggered a loss of collagen (Figure 6a), and treatment with either the MMP or broad-spectrum metalloproteinase inhibitor abolished IL-1-induced collagen degradation. Unstimulated controls exhibited low rates of collagen release, and IL-1-stimulation elevated release from day 6 to 12 (Figure 6b). Addition of the aggrecanase-1,2-selective inhibitor did not affect collagen release, consistent with its specific effects on ADAMTS-4 and -5, which have no proteolytic activity against collagen. Interestingly, this pattern is in contrast to articular cartilage, where aggrecanase inhibition delayed the onset of IL-1-induced collagen destruction [26,27] presumably by blocking access of the MMPs to the collagen rather than through direct MMP inhibition. Treatment with the MMP-selective or broad spectrum metalloproteinase inhibitors potently inhibited IL-1-induced collagen depletion in meniscal explants, confirming that MMPs were primary mediators of collagen destruction in this culture system.

### Inhibition of IL-1-induced loss of mechanical properties in the immature meniscus

**Figure 7:**
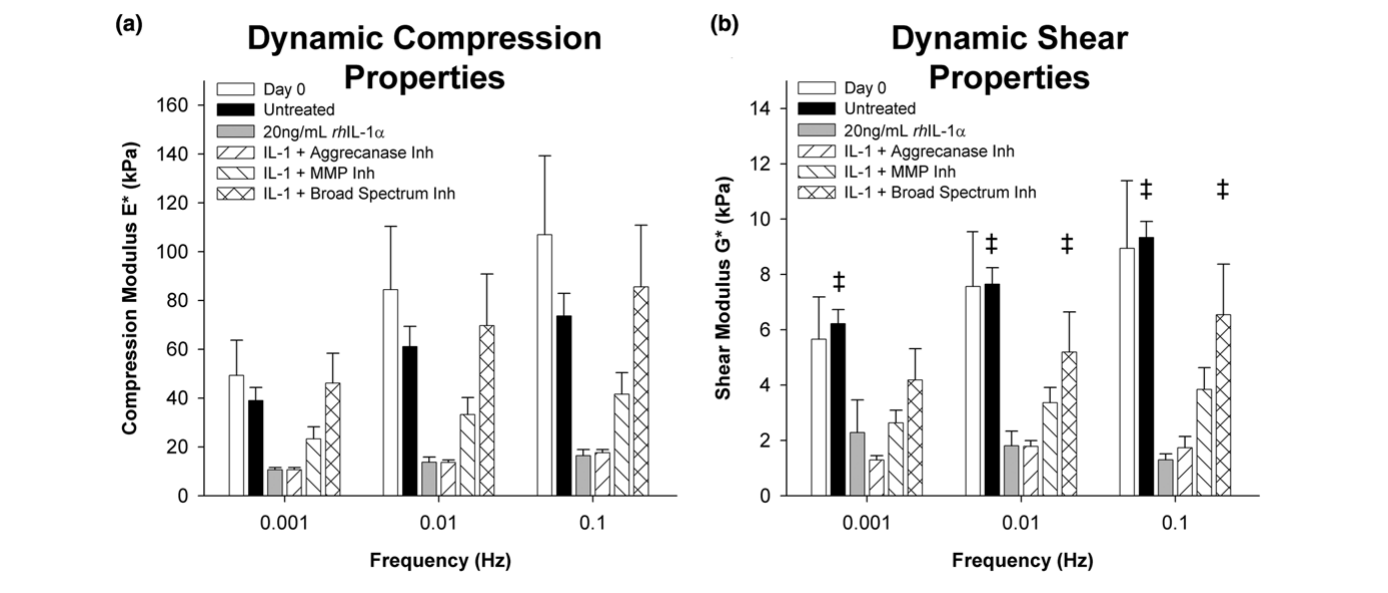
Inhibition of IL-1-induced loss of meniscal explant dynamic (a) compression and (b) shear moduli by metalloproteinase inhibitors. Explants from day 0 and 12 were tested in oscillatory unconfined compression and torsional shear. Data are mean + standard error of the mean, n = 6. ‡ indicate *P *< 0.05 vs. IL-1. MMP = matrix metalloproteinase.

In both unconfined compression and torsional shear tests, day 0 tissue exhibited trends of increasing modulus with frequency, and these trends were maintained in unstimulated controls following 12 days of culture (Figure 7). Significantly, the compression and shear properties of these explants were maintained over 12 days of culture, despite evidence of persistent aggrecanolysis (Figures 4 and 5), suggesting that generation of NITEGE in the immature bovine meniscus is compatible with maintenance of this tissue's mechanical properties (perhaps due to contributions by proteoglycans other than aggrecan). Stimulation with IL-1 led to reductions in both compression and shear moduli, and the aggrecanase- and MMP-selective inhibitors did not significantly ameliorate these losses. IL-1-stimulated explants cultured with the broad-spectrum inhibitor, however, had significantly higher compression and shear moduli than IL-1-only controls.

## Discussion

The results of this study demonstrate regional variations in the accumulation of aggrecanase-generated aggrecan fragments in the developing bovine meniscus. In articular cartilage, the NITEGE neoepitope is indicative of tissue degeneration and is essentially undetected in immature tissue. In contrast, extensive proteolytic processing of aggrecan, yielding products found in degenerative articular cartilage, appears to be normal in the developing meniscus. NITEGE was abundant in the middle and outer zones of immature meniscal fibrocartilage (as previously reported [13]) and was steadily released from middle zone explants over 12 days in culture. Interestingly, safranin-O staining is more uniformly distributed and aggrecan gene expression is higher in menisci from mature animals than from immature animals, suggesting that these regional variations in aggrecan density and turnover are dependent on developmental stage [3]. Although synthesis of proteoglycans can be regulated at the transcriptional (mRNA) level, regional variations in immature meniscus sGAG content may be due in part to regional differences in aggrecanase activity.

Blood vessels were readily detected in the middle and outer regions of the immature meniscus, but absent in the inner region, suggesting a spatial relation between vascular supply and aggrecan destruction in this tissue. It has been suggested that the vasculature and greater oxygen tension of the outer meniscus can suppress cartilage-like matrix formation [41], and preservation of a chondrogenic phenotype similar to inner region cells may be contingent upon a hypoxic environment [42]. Further, age-related spatiotemporal changes in meniscal vascular density are consistent with the inversely related changes in sGAG localization, because the early post-natal human meniscus is vascularized throughout while the mature (over 20 years) meniscus is vascularized only in the outer 25 to 33% [43]. Aggrecanolysis may thus be regulated by access to the meniscal vascular supply or, conversely, serve to regulate vascular persistence in the meniscus. Interestingly, the fat pads of developing mice and the flexor tendons from young and mature bovines were also found to contain NITEGE-bearing aggrecan [44,45], suggesting that degradation of aggrecan has physiologic functions in other vascularized tissues as well.

Regional differences in mechanical loading may contribute to the spatial pattern of aggrecanolysis in the meniscus. Finite element models predict substantial regional variations in complex combinations of compressive, tensile, and shear strains during physiologic loading [46]. Regions of bovine flexor tendon that undergo primarily tensile loading *in vivo *synthesized less high molecular weight proteoglycan than explants of compressed tendon [47]. In addition, cells from the inner and outer regions of the meniscus exhibited distinct biosynthetic responses to biaxial mechanical loading [48]. It is likely that interactions between soluble and mechanical factors regulate aggrecanolysis in the meniscus, and may contribute to the development of a mechanically appropriate ECM. Extensive aggrecan cleavage may be necessary for the assembly of large tension-bearing collagen fibrils in the middle and outer regions of the meniscus, especially if proper alignment of the collagen fibrils is a cell adhesion-dependent process. Aggrecan inhibits nerve outgrowth partly due to the presence of sGAG, and perineuronal nets are composed of aggrecan [49,50]. It is possible that meniscal cells, particularly in the middle and outer regions, would not attach to the ECM or retain a stellate morphology in the presence of full-length aggrecan. There is a direct relation between rounded cell morphology and sGAG abundance in articular cartilage and in the developing meniscus. Maintenance of the stellate morphology may be essential for fibrocartilage-specific mechanotransduction pathways, which in turn regulate ECM remodeling.

IL-1-stimulated menisci stained intensely for NITEGE in the inner and middle regions, where sGAG density was initially highest. These results may actually underestimate the abundance of NITEGE generated in the meniscus because the surface zones of this tissue (removed for the explant experiment) appear to have robust aggrecanase activity (Figure 3), consistent with previously reported elevation of MMP-3 expression, aggrecanase activity, and sGAG release from IL-1-treated surface-zone meniscal fibrocartilage from mature bovines [51]. The sGAG release observed in the current explant study was likely due to the degradation and/or release of aggrecan as well as other proteoglycans. Because the G1-NITEGE fragment of aggrecan, abundant in the immature meniscus, lacks sGAG attachment regions, the contribution of this species to sGAG content and release would be negligible. sGAG was detected in conditioned media throughout the culture period, but high molecular weight aggrecan G1 species were absent from conditioned media collected late in the experiment, suggesting that other proteoglycans contributed to the release of sGAG, particularly at later stages of degradation. IL-1 induced the loss of decorin in this study, and meniscal cells from the immature rabbit express mRNA for another large proteoglycan, versican [52]. In addition, decorin, biglycan, fibromodulin, and versican have been immunolocalized in fibrocartilage of the intervertebral disc [53], suggesting that a variety of large and small proteoglycans contribute to the pool of sGAG in the ECM of fibrocartilages. It is important to note that sGAG-bearing aggrecan may be retained within fibrocartilage independent of G1-hyaluronan interactions. Vogel and colleagues detected sGAG-bearing aggrecan lacking the G1 domain in regions of tendon that undergo tensile loading [8], and we have recently reported that multiple aggrecan G3 fragments accumulate in immature meniscal fibrocartilage [40]. The G3 domain can bind ECM proteins such as fibulin and tenascin-C [54,55], and such interactions could facilitate retention of aggrecan in the meniscus.

The mechanical functions of meniscal fibrocartilage depend on the composition, integrity, and organization of its ECM [56]. Explanted meniscal fibrocartilage stimulated with IL-1 for 12 days contained 44% and 67% of the sGAG and collagen, respectively, in day 0 tissue. Correspondingly, the depleted tissue retained only 15 to 40% of the shear stiffness and 15 to 22% of the compressive stiffness of the fresh tissue. Explants treated with the MMP inhibitor retained 100% of the initial collagen but only 63% of the initial sGAG. This reduction in sGAG was associated with a 53 to 57% reduction in shear stiffness and a 53 to 61% reduction in compressive stiffness, indicating that despite comprising a quantitatively minor ECM constituent (1 to 3% by dry weight) [57], proteoglycans contribute substantially to the shear and compression properties of immature bovine meniscal fibrocartilage.

Aggrecanases were mediators of aggrecan proteolysis in the immature meniscus, as evidenced by an abundance of NITEGE in tissue extracts. In contrast, MMPs did not appear to contribute substantially to aggrecanolysis in this system because characteristic MMP-generated aggrecan fragments (identified by G1 bands migrating at about 55 kDa) were not detected in tissue extracts or conditioned media. MMPs did, however, appear to play a strong role in sGAG release from meniscal explants. IL-1-induced loss of sGAG was significantly reduced by treatment with an MMP-selective inhibitor or a broad-spectrum (aggrecanase and MMP) inhibitor but not with an aggrecanase-1,2-selective inhibitor. The selectivities of these inhibitors for aggrecanases other than aggrecanase-1 or -2 (e.g., ADAMTS-1, -8, -9, -15, -16, and -18) are unknown, making it difficult to fully distinguish between the contributions of MMPs and aggrecanases other than ADAMTS-4 or -5 to the release of sGAG in these experiments. However, in a similar model of tissue resorption with age- and species-matched articular cartilage, ADAMTS-4 and -5 mediated 90% of the observed aggrecanase activity and loss of sGAG [58]. ADAMTS-5 was found to be the dominant aggrecanase in mouse models of arthritis [23,24], and a recent report indicates that ADAMTS-8, -15, -16, and -18 are expressed at very low levels in mouse cartilage [59].

MMPs may regulate sGAG release by disrupting the aggrecan-hyaluronan aggregate. IL-1-induced depolymerization of hyaluronan by free radicals or hyaluronidases, shown to occur in articular cartilage [60,61], may be influenced indirectly by MMP activity (or the inhibitors used here). Link protein, which stabilizes the interaction between aggrecan G1 and hyaluronan, is a substrate for MMP-3 [62], and CD44, a hyaluronan receptor, is a substrate for MMP-14 [63]. Release of sGAG from IL-1-stimulated fibrocartilage may be further regulated by MMP-mediated collagen degradation. The organized bundles of collagen fibrils in fibrocartilage may demarcate a metabolic pool of aggrecan for which release is rate-limited by reaction of MMPs with collagen. Akin to the fascicular structures present in tendon and muscle, these structures appear to contain relatively thick fibril bundles. Presumably, MMP-mediated disruption of the fascicular structures would enhance diffusion of collagen-associated aggrecan out of these structures. MMPs thus appear to serve an indirect, but quantitatively significant, role in the release of aggrecan and sGAG from the cytokine-stimulated immature meniscus.

## Conclusions

Clinical and basic research over the past two decades has demonstrated the importance of the menisci in the maintenance of healthy knee joint mechanics. Whereas articular cartilage has been the subject of intense research in the context of joint degeneration, subtle changes in meniscal composition may be more closely associated with the true 'onset' of knee osteoarthritis [52]. The results of this study indicate that meniscal fibrocartilage exhibits regional variations in aggrecan turnover that may be important for normal development of the tissue. In addition, whereas aggrecanases were the primary mediators of aggrecanolysis in the meniscus, the release of sGAG from this tissue appears to be mediated primarily by MMPs or aggrecanases other than ADAMTS-4 and -5. These results may have relevance for the clinical management of joint disease, as understanding the ECM remodeling events driving normal development and cytokine-induced degradation of the meniscus will be helpful in developing strategies for repairing injured and diseased meniscal fibrocartilage.

## Abbreviations

ADAMTS: a disintegrin and metalloproteinase with thrombospondin motifs; DAPI: 4',6-diamidino-2-phenylindole dihydrochloride; DMEM: Dulbecco's Modified Eagle's Medium; DMMB: dimethylmethylene blue; ECF: enhanced chemifluorescence; ECM: extracellular matrix; FITC: fluoroscein isothiocyanate; HEPES: N-(2-hydroxyethyl)-piperazine-N'-2-ethanesulfonic acid; IL-1: interleukin-1; MMP: matrix metalloproteinases; NBF: neutral buffered formalin; NEAA: non-essential amino acids; PBS: phosphate buffered saline; rh: human recombinant; sGAG: sulfate glycosaminoglycans; TNF: tumor necrosis factor.

## Competing interests

FZ was an employee of Roche-Palo Alto when the studies were performed. All authors declare that they have no competing interests.

## Authors' contributions

CGW carried out the culture studies and performed the biochemical assays and mechanical testing. EJV participated in tissue isolation and the immunofluorescence studies. FZ contributed inhibitors and technical support for the use of the inhibitors. JDS advised on the characterization of aggrecan cleavage products. CGW and MEL conceived of and designed the studies and performed the statistical analysis. CGW, MEL, and JDS wrote the manuscript. All authors read and approved the final manuscript.
